# What transposable elements are differentially translated in lung cancer?

**DOI:** 10.1186/2051-1426-1-S1-P139

**Published:** 2013-11-07

**Authors:** Wan R Yang, Eric W Mills, Nemanja Rodic, Nicholas T Ingolia, Jef D Boeke, Hyam I Levitsky, Kathleen H Burns

**Affiliations:** 1 Pathology, Johns Hopkins University School of Medicine, Baltimore, MD, USA; 2 Oncology, Johns Hopkins University School of Medicine, Baltimore, MD, USA; 3 Embryology, Carnegie Institution for Science, Baltimore, MD, USA; 4 Molecular Biology and Genetics, Johns Hopkins University School of Medicine, Baltimore, MD, USA

## 

Transposable element (TE) expression is generally silent in somatic tissues, due to significant genomic methylation and other redundant methods of silencing. Cancer tissues, however, exhibit a marked decrease in methylation throughout the genome, which can result in de-repression of transposable element transcription. Because of this phenomenon, TEs may be tumor-specific antigens for use as potential biomarkers and vaccine targets. Lung cancer in particular is in dire need of early screening tools and treatment; the five-year survival rate is 16%. Ideal biomarkers and vaccine targets for lung cancer would be proteins or polypeptides that can be recognized by the immune system. However, TEs are subject to multiple post-transcriptional silencing mechanisms, such that increased hypomethylation does not necessarily result in increased polypeptide expression. Although evidence of increased transposable element RNAs and proteins in cancer tissues (in particular LINE-1 and HERV-K) exist in the literature, translation of other retrotransposon-encoded intermediates and other repetitive transcripts has not been thoroughly investigated. To this end we selectively sequenced translated TE sequences in a conditional mouse model of lung cancer, using ribosomal profiling. Comparing ribosomal footprints of healthy wild-type and transgenic cancerous lung tissues allows us to identify differentially translated elements.

